# The role of TCGA molecular classification in clear cell endometrial carcinoma

**DOI:** 10.3389/fonc.2023.1147394

**Published:** 2023-06-29

**Authors:** Xinyue Tang, Yuanjing Hu

**Affiliations:** ^1^ Graduate School, Tianjin Medical University, Tianjin, China; ^2^ Department of Gynecological Oncology, Tianjin Central Hospital of Obstetrics & Gynecology, Tianjin, China

**Keywords:** clear cell endometrial carcinoma, TCGA, molecular classification, prognosis, adjuvant therapy

## Abstract

Clear cell endometrial carcinoma (CCEC) represents a relatively rare and heterogeneous entity. Based on The Cancer Genome Atlas (TCGA) molecular classification, the risk stratification and management of endometrial cancer (EC) have been improved. Although the relationship of CCEC with the TCGA classification is less well understood, data has emerged to suggest that molecular classification plays an important role in the prognosis and management of CCEC. Most of patients with CCEC are characterized by p53abn or NSMP type and the prognosis of these patients is poor, whereas those with MMRd or POLEmut seem to have a favorable prognosis. Adjuvant therapy is recommended in CCEC with p53abn and NSMP. Advanced/recurrent CCEC with MMRd benefit much more from immune checkpoint inhibitors after the failure of platinum-based chemotherapy. In addition, bevacizumab plus chemotherapy upfront seems to improve outcomes of advanced/recurrent patients whose tumors harbored mutated TP53, including CCECs with p53abn. Further studies which exclusively recruit CCEC are urgently needed to better understand the role of molecular classification in CCEC. This review will provide an overview of our current understanding of TCGA classification in CCEC.

## 1 Introduction

Clear cell endometrial cancer (CCEC) is an uncommon but aggressive histologic type that accounts for 1%-6% of endometrial cancer (EC), and characterized by poorer prognosis and chemotherapy resistance (1). A clear cell endometrial carcinoma usually features HNF1β positive, Napsin A positive, WT1 negative and estrogen receptor (ER)/progesterone receptor (PR) negative (2). Owing to the rarity of clear cell endometrial cancer, several features regarding CCEC are still unclear.

Traditionally, based on biological and clinical parameters, endometrioid endometrial cancer is considered as “type I” EC and accounts for 80% of EC, whereas non-endometrioid (i.e. serous and clear cell) histology tumors has been regarded as “type II” EC since it is not estrogen-related and has poor prognosis (3–5). However, CCEC overlaps with endometrioid and serous carcinoma in many features: morphological, immunohistochemical, molecular and prognostic, so the new classifications are needed. In 2013, The Cancer Genome Atlas (TCGA) classified EC into 4 subtypes: POLE ultramutated (POLE), microsatellite instability hypermutated (MSI), copy-number low (CNL) and copy-number high (CNH) (6). As surrogate markers of the TCGA molecular subtypes, the Proactive Molecular Risk Classifier for Endometrial Cancer (ProMisE) subdivided different endometrial carcinomas into four prognostic molecular subgroups: POLE-mutated (POLEmut), mismatch-repair- deficient (MMRd), TP53-wild-type (NSMP) and TP53-abnormal (p53abn), and identified 4 molecular subtypes with distinct prognostic outcomes (7–9). Notably, clear cell endometrial carcinomas were not involved in the study. Unlike EC, the relationship between CCEC and TCGA classification has not been fully elucidated. In the present review, we tried to provide a comprehensive overview of the role of molecular classification in prognosis and management of CCEC.

## 2 The value of TCGA classifier in clear cell endometrial carcinoma

Since TCGA classification of EC was proposed, several groups have described the molecular classification of CCEC. CCEC was found within all four molecular subtypes and encompassed a wide range of clinical outcomes (4, 10, 11). Results are consistent across different reports, and demonstrated that the most prevalent subgroups were the p53abn and NSMP subgroups, while the MMRd and POLEmut subgroups were less common (9, 10, 12). A recently published meta-analysis suggested that POLEmut, MMRd, p53abn and NSMP accounted for about 3.5%, 11.4%, 35.1%, and 50% of patients with pure CCEC, respectively, while MMRd subgroup constituted the majority of mixed CCEC, accounting for about 50% of mixed CCECs (4). Women with p53abn and NSMP CCECs were older than women with MMRd and POLEmut subtypes. As a unique subgroup, the NSMP CCECs showed distinct clinical and pathological features, in particular older age, lower BMI, more aggressive clinical course and absent or minimal ER expression, compared to other NSMP ECs (10). In terms of prognosis, MMRd CCECs had a favorable prognosis with a 5-year OS >95%, while the prognosis of NSMP CCECs did not significantly differ from that of p53abn CCCs, with a 5-year OS <50% (4). In this review, none of the POLEmut patients died, which meant POLEmut CCCs conferred favorable prognosis. Other studies have also come to the conclusion consistent with this study, namely that patients with MMRd or POLEmut have better outcome than those with p53abn and NSMP (10, 11). Interestingly, some recent studies have analyzed the relationship between TCGA groups and classic prognostic factors (myometrial invasion, lymphovascular space invasion (LVSI)) in ECs (including CECCs) (13). LVSI was not associated with an increased risk of tumor recurrence or progression and death from disease in POLE-mt ECs, while it appeared as an independent predictor of poor outcome in the MSI group (14–16). Deep myometrial invasion did not appear as an independent prognostic factor for OS in EC patients; instead, it seemed to affect the risk of recurrence independently from the TCGA groups (17).

### 2.1 Adjuvant therapy

According to the NCCN guidelines, CCEC is considered a high-risk histologic type of EC and requires adjuvant therapy in most case. Even in early-stage CCEC, the risk of recurrence is still high and adjuvant chemotherapy mitigates the risk of distant metastases (18), however, in terms of decision-making regarding adjuvant treatment, the role of molecular classification is not still elaborated (19). Significantly, molecular classification has been incorporated into the ESGO/ESTRO/ESP guidelines as fundamental integrated information for prognostic risk group stratification and for tailoring adjuvant therapy in EC patients (3). Herein, we extracted CCEC-related descriptions from the updated risk stratification system (**Table 1**). According to ESGO/ESTRO/ESP guidelines, adjuvant treatment could be omitted for stage I/II CCEC patients with POLEmut of low-risk group, while chemotherapy +/− radiotherapy is recommended for stage I-IVA CCEC patients with p53abn and myometrial invasion of high-risk group. Due to the lack of randomized trials, the potential benefit of adjuvant therapy for CCEC patients of intermediate-risk group is unclear, consequently, the recommendation for adjuvant treatment or observation should be considered on a case-by-case basis following multidisciplinary discussion (3). Of note, CCECs with the molecular profile MMRd or NSMP are not allocated to the prognostic risk group in the ESGO/ESTO/ESP guidelines as only limited data were available for their prognostic relevance. Thus, for these patients, inclusion into prospective registries is recommended.

**Table 1:** Risk group related to clear cell endometrial carcinoma extracted from the ESGO/ESTRO/ESP guidelines.

Risk group	Description related to CCEC^a^
Low risk	Stage I/II POLEmut CCEC; for stage III POLEmut cancers^b^
Intermediate risk	Stage IA and/or p53-abn CCEC without myometrial invasion and no or focal LVSI
High-intermediate risk	None
High risk	All stage CCEC with p53-abn and myometrial invasion

CCEC, clear cell endometrial carcinoma; EC, endometrial cancer; LVSI, lymphovascular space invasion; p53-abn, p53-abnormal; POLEmut, polymerase epsilon-ultramutated.

^a^Stage III-IVA if completely resected without residual disease; table does not apply to stage III-IVA with residual disease or for stage IV.

^b^POLEmut stage III might be considered as low risk. Nevertheless, currently there are no data regarding safety of omitting adjuvant therapy.

PORTEC-3 trial explored the benefit of combined adjuvant chemotherapy and EBRT(CTRT) versus EBRT alone in patients with high-risk EC (including CCEC) (20, 21). However, there is substantial interobserver variability in assessment of pathologic factors that define high-risk, so it remains a challenge to identify patients who will benefit from chemotherapy (22). In contrast, the molecular classification of EC is characterized by higher reproducibility. Following PORTEC-3 trial, León-Castillo, A., et al. used tissue samples from the PORTEC-3 clinical trial to investigate the prognostic relevance of the molecular classification and the relationship between the molecular subgroups and benefit from adjuvant CTRT in patients with high-risk EC (23). This study showed that patients with p53abn EC had a highly significant benefit from CTRT versus RT alone and patients with NSMP EC had a trend toward benefit from CTRT. Considering that p53abn and NSMP represent the majority of CCEC (4, 12), the above study is of great significance to guide the adjuvant treatment of CCEC. Although NSMP molecular subtype is not enrolled into the updated risk stratification system of ESGO/ESTO/ESP guidelines, it might be included in a high-risk category due to its aggressive features with the highest proportion of LVSI, deep myometrial invasion, node positive and advanced stage (III/IV) disease (10). The prognosis of NSMP CCECs seems not to significantly differ from p53abn CCECs, supporting a similar management for these two groups of patients.

It needs to be emphasized that patients with mixed endometrioid and clear cell carcinoma are characterized by MMRd (24). Similarly, Travaglino, A., et al. also pointed out that MMRd subgroups constituted the majority of mixed CCEC, accounting for about 50% of mixed CCECs (4). Therefore, it makes sense to explore adjuvant therapy strategies for CCEC with MMRd. However, only limited data are available for CCEC with MMRd. Molecular analysis of the PORTEC-3 trial suggested no, or limited benefit of adding chemotherapy in patients with MMRd EC (23). According to the recently published meta-analysis, for CCEC with MMRd, 5-year OS was 95.7 ± 4.3% in the main analysis and 90.9 ± 6.7% in the pure CCEC subgroup, and none of the MMRd patients died in the mixed CCEC group (4). The result supported that MMRd CCECs had a favorable prognosis, and MMRd CCECs might be included in a lower risk category. Therefore, further prospective data are needed to better define prognosis and role of adjuvant therapy in the MMRd group.

Significantly, the integration of molecular signature and clinicopathological factors would provide a more tailored management for ECs (25–27). An integrated clinicopathologic and molecular risk profile was established for EC with HIR features, separating them in favorable, intermediate and unfavorable groups, each with a clearly different prognosis (25). in this study it was shown that L1 cell adhesion molecule (L1CAM) overexpression was significant risk factors for both pelvic and distant recurrences, and within the NSMP group, β catenin (CTNNB1) was also found to be prognostic for distant recurrence. To evaluate the clinical role of this molecular-integrated risk profile in the determination of adjuvant treatment in patients with HIR EC, the PORTEC-4a study was initiated in 2016 (26, 28). Women with a favorable profile (POLE mutation, or NSMP without CTNNB1 mutations) were observed after surgery; women with an intermediate risk profile (mismatch repair-deficient (MMRd) or NSMP with CTNNB1 mutations) received adjuvant VBT; and women with any of the unfavorable risk factors (substantial LVSI, TP53 abnormal immunohistochemical staining or L1CAM overexpression) were treated with EBRT (26, 28). The primary endpoint of PORTEC-4a (NCT03469674) is vaginal recurrence, and the results are worth looking forward to. It is necessary to remark that these studies only included patients with endometrioid carcinoma, but it paves the way for future exploration of the integration of molecular signature and clinicopathological factors in CCEC population.

### 2.2 Treatment of recurrent/advanced CCEC

Patients with recurrent/advanced disease are characterized by poor prognosis, with 5-year OS rates of 20%-25% (29). The treatment of patients with recurrent and progressive EC should be guided by several features, including the patient’s condition, extent of the disease, prior therapies and molecular profile, and should always require a multidisciplinary approach which includes surgery, radiotherapy (RT), and chemotherapy (ChT). For advanced/recurrent disease not amenable to surgery and/or RT, the standard approach remains ChT or hormonal therapy. Currently, carboplatin AUC 5-6 plus paclitaxel 175 mg/m^2^ every 21 days for six cycles should be considered the first-line therapy for recurrent or metastatic EC (3). In addition, some novel treatments are under constant exploration.

#### 2.2.1 immune checkpoint inhibitors

NCCN and ESGO/ESTO/ESP guidelines have recommended several immune checkpoint inhibitors as a second-line treatment for recurrent/metastatic EC with MMRd (3, 19). Le, D.T., et al. have confirmed that the large proportion of mutant neoantigens in mismatch repair-deficient (MMRd) cancers made them sensitive to immune checkpoint blockade, regardless of the cancers’ tissue of origin (30). In 2017, the Food and Drug Administration (FDA) approved pembrolizumab [anti-programmed cell death protein 1 (PD-1)] for treatment of advanced MSI-H or MMRd solid tumors. Given that 25%-30% of primary ECs are MMRd, indicating immune dysregulation, several immune checkpoint inhibitors have been approved for treatment of specific ECs. The KEYNOTE-158 clinical trial of pembrolizumab enrolling patients with MSI-H/dMMR advanced noncolorectal cancer who experienced failure with prior therapy confirmed the durable antitumor activity on EC population (including CCEC) (31, 32). Based on the KEYNOTE-158 trial, On March 21, 2022, FDA approved pembrolizumab, as a single agent, for patients with advanced endometrial carcinoma that is microsatellite instability-high (MSI-H) or mismatch repair deficient (MMRd), who have disease progression following prior systemic therapy in any setting and who are not candidates for curative surgery or radiation (33). The activity and safety of dostarlimab, an anti-PD-L1 (programmed death-ligand 1) agent, were analyzed in the GARNET trial (34). This ongoing phase Ib study has enrolled 104 patients with MMRd EC. Of these, 71 had measurable disease at baseline and 6 months follow-up and were included in the primary analysis. The confirmed ORR was 42.3% (a confirmed complete was seen in 12.7% patients; a partial response was seen in 29.6% patients) (34). In light of these results, FDA granted accelerated approval to dostarlimab-gxly for adult patients with MMRd recurrent or advanced endometrial cancer that has progressed on or following a prior platinum-containing regimen (35). A phase III trial (KEYNOTE-775) including 827 EC patients (697 with pMMR disease and 130 with dMMR disease) with previously treated showed that pembrolizumab plus lenvatinib led to significantly longer OS (pMMR population: HR 0.68, 95%CI 0.56-0.84, P<0.001; overall: HR 0.62, 95%CI 0.51-0.75, P<0.001) and PFS (pMMR population: HR 0.60, 95%CI 0.50-0.72, P<0.001; overall: HR 0.56, 95% CI 0.47-0.66, P<0.001) than chemotherapy of the treating physician’s choice (doxorubicin or paclitaxel) with advanced EC (36). Based on the results, FDA approved pembrolizumab in combination with lenvatinib for patients with advanced endometrial carcinoma that is not MSI-H/dMMR, who have disease progression following prior systemic therapy in any setting and are not candidates for curative surgery or radiation (37). However, only 47 CCECs were included in the KEYNOTE-775.Several ongoing trials are evaluating the activity of various checkpoint inhibitors in patients with recurrent/advanced CCEC. The information from the clinicaltrials.gov database is shown in **Table 2**.

**Table 2:** Ongoing trials of immune checkpoint inhibitors in clear cell endometrial carcinoma.

ClinicalTrials.gov Identifier	Agent	Phase	Participants	Primary endpoint	Estimated completion date
NCT03603184	Atezolizumab+ Paclitaxel/Carboplatin	III	Patients with advanced/recurrent EC, including CCEC	Progression-free survival and overall survival	December, 2023
NCT03914612	Pembrolizumab+ Carboplatin/Paclitaxel	III	Patients with advanced/recurrent MMRd EC,including CCEC	Progression-free survival	June, 2023
NCT05419817	Pembrolizumab+ Sitravatinib	II	Patients with recurrent EC and other solid tumors with MMRd,including CCEC	Objective response	December, 2026
NCT05112601	Nivolumab+ Ipilimumab	II	Patients with advanced/recurrent MMRd EC,including CCEC	Progression-free survival	April, 2026
NCT04463771	Retifanlimab+ Other therapies	II	Patients with advanced/metastatic EC, including CCEC	Objective response rate	June, 2025
NCT03367741	Nivolumab+ Cabozantinib	II	Patients with advanced,recurrent or metastatic EC,including CCEC	Progression-free survival	October, 2023
NCT03241745	Nivolumab	II	Patients with MMRd/hypermutated uterine cancer,including CCEC	Progression-free survival	August, 2023
NCT02715284	Dostarlimab	I	Patients with advanced solid tumors,including EC which includes CCEC	Number of treatment emergent AEs(TEAEs)	October, 2027
NCT05092373	Atezolizumab+ Cabozantinib/Nab-paclitaxel	I	Patients with advanced solid tumors,including EC which includes CCEC	To assess the safety and tolerability of TTF, including the maximum tolerated dose (MTD)	September, 2026
NCT04272034	INCB099318	I	Patients with advanced solid, including CCEC	Number of treatment emergent AEs(TEAEs)	June, 2024

MMRd, mismatch repair deficient; EC, endometrial cancer; CCEC, clear cell endometrial carcinoma; AEs, adverse events; TTF, tumor treating fields.

In conclusion, the MMRd subtype plays an important role in the application of immunotherapy in advanced/recurrent CCEC. Similar to MMRd ECs, POLEmut ECs are also characterized by an extensive immune infiltrate, and their immunogenicity is thought to be the cause of their favorable prognosis (38, 39), however, few clinical trials that study the role of immune checkpoint inhibitors in POLEmut EC have been reported. Further evidence is needed to clarify the benefit of adding immunotherapy in patients with recurrent/progressive CCEC according to molecular classification.

#### 2.2.2 targeted therapy

To some extent, molecular classification also plays a role in the targeted therapy of advanced/recurrent EC, including CCEC. According to NCCN guidelines, carboplatin/paclitaxel/bevacizumab could be considered as front-line systemic therapy for patients with advanced/recurrent EC. However, GOG86p trial, one of the first trials combining a targeted agent (either bevacizumab or temsirolimus) with standard chemotherapy for high risk or recurrent EC, showed no PFS benefit compared with historical controls, namely the carboplatin-paclitaxel arm of trial GOG209 (40, 41). Recently, based on molecular classification, Leslie, K.K., et al. performed an exploratory analysis to assess TP53 mutational status in patients from GOG86P and determined the implications on clinical outcomes (42). This exploratory study suggested that combining chemotherapy with bevacizumab, but not temsirolimus, might enhance PFS (HR 0.48;95%CI 0.31,0.75) and OS (HR 0.61;95% CI 0.38, 0.98) for patients whose tumors harbor mutant p53, whereas patients with P53wt did not have a markedly different PFS or OS on the bevacizumab arms compared to the temsirolimus arm. From a mechanistic perspective, the reason why p53 mutation is related to improvement in outcomes in response to bevacizumab may be the described link between the p53 protein and VEGF: wild type p53 protein inhibits transcription of angiogenic factors such as VEGF-A. It is reported that mutations in TP53 that negatively impact p53 wild type transcriptional activity may alleviate transcriptional repression of VEGF-A, resulting in higher expression of the direct target of bevacizumab (42, 43). To our knowledge, CCEC with p53abn accounts for 35% of all CCECs, and for those, bevacizumab combined with chemotherapy may be a good option. Limitedly, a chemotherapy-only reference arm was not included in the GOG-86P trial design, and sequencing was not performed on subjects from historical controls GOG-209 (42). Another potential limitation of this study is that only 16 CCEC patients were included in this study, and TP53 mutational analysis was available for only 7 of them. Future trials are needed to compare bevacizumab plus chemotherapy with chemotherapy alone in CCEC patients with p53abn.

Furthermore, since HER-2 overexpression has been described in the p53abn CCEC, it is possible that the subgroup may be sensitive to anti-HER-2 targeted therapy (27, 44). Additionally, a subset of p53abn ECs (including CCECs) shows high DNA damage and high PARP-1 expression, offering the possibility of using PARP-inhibitors to treat these cases (27, 45).

Of note, no data are available specifically for CCEC, either on immune checkpoint inhibitors or on targeted therapy. Successfully combining targeted agents and immunotherapy with molecular classification is an important future goal for recurrent/advanced CCEC therapy.

## 3 Conclusions

Herein, we summarize the role of molecular classification in the management and prognosis of clear cell endometrial cancer (CCEC) which represents an uncommon disease entity, with different characteristics from endometrioid and other non-endometrioid cancers. Theoretically, adjuvant therapy could be omitted in patients with stage I/II CCEC harboring POLE mutation, whereas adjuvant therapy is recommended in patients with NSMP and p53abn CCEC. With respect to MMRd CCEC, recommendations for adjuvant therapy are unclear. Immunotherapy seems to be the more promising treatment option for patients with advanced or recurrent CCEC characterized by MMRd. In terms of prognosis, CCECs with p53abn and NSMP account for a large majority of all CCECs and have poor clinical outcomes, while those with MMRd or POLEmut have very favorable outcomes. Clinical outcomes of CCEC are different from what has been reported previously, where review of EC in which molecular subtype classification had been applied revealed that POLE-mutated EC has a favorable prognosis, p53abn EC has a poor prognosis, and MMRd and NSMP EC have the intermediate prognosis. Thus, NSMP CCEC appear to be a distinct clinicopathological entity within the larger group of NSMP ECs. Moreover, the integration of molecular signature with pathological factors and genomic profiling would ensure a more tailored management of patients in accordance with the principles of the precision medicine. To date, the number of patients with CCEC is relatively small and few studies have focused exclusively on CCEC in the context of the TCGA classification, therefore, further studies that focus specially on CCEC are necessary, and future clinical trials which include molecular classification subgroups and specific targeted treatments in their design are also needed.

## Author contributions

YH conceived the project. All authors reviewed the literature and drafted the article. YH revised the manuscript. All authors contributed to the article and approved the submitted version.
